# Genetic Studies of Inflammatory Bowel Disease-Focusing on Asian Patients

**DOI:** 10.3390/cells8050404

**Published:** 2019-05-01

**Authors:** Sung Chul Park, Yoon Tae Jeen

**Affiliations:** 1 Division of Gastroenterology and Hepatology, Department of Internal Medicine, Kangwon National University School of Medicine, Chuncheon 24289, Korea; schlp@hanmail.net; 2 Division of Gastroenterology and Hepatology, Department of Internal Medicine, Korea University Anam Hospital, Korea University College of Medicine, 73, Inchon-ro, Seongbuk-gu, Seoul 02841, Korea

**Keywords:** genetics, inflammatory bowel disease, Crohn’s disease, ulcerative colitis

## Abstract

The pathogenesis of inflammatory bowel disease (IBD) is not well-understood; however, increased and persistent intestinal inflammation, due to inappropriate immune responses that are caused by interactions between genetic factors, gut microbiota, and environmental factors, are thought to lead to IBD. Various studies have identified more than 240 genetic variants related to IBD. These genetic variants are involved in innate and adaptive immunity, autophagy, defective bacterial handing, interleukin-23 and 10 signaling, and so on. According to several epidemiological and clinical studies, the phenotypes and clinical course of IBD differ between Asians and Europeans. Although the risk loci for IBD typically overlap between Asians and Westerners, genetic heterogeneity has been detected in many loci/genes, such as *NOD2/CARD15*, *TNFSF15* and human leukocyte antigen, contributing to the risk of IBD. Thus, although common pathways exist between Westerners and Asians in the development of IBD, their significance may differ for individual pathways. Although genetic studies are not universally applicable in the clinical field, they may be useful for diagnosing and categorizing IBD, predicting therapeutic responses and toxicity to drugs, and assessing prognosis by risk modeling, thereby enabling precision medicine for individual patients.

## 1. Introduction

Inflammatory bowel disease (IBD) is a disease that causes chronic inflammation of unknown origins in the intestine. It is divided into ulcerative colitis (UC) and Crohn’s disease (CD). Some patients have a mild clinical course. However, a considerable number of patients undergo repetitive improvement and deterioration, with some requiring surgical treatment [1]. The mechanism of IBD is not well-known yet. However, previous studies have suggested that intestinal inflammation is increased or persists because of inappropriate immune responses caused by interactions between genetic factors, gut microbiota, and environmental factors, leading to the development of IBD. The results of previous studies support the speculation that genetic factors are involved in IBD, keeping in view the fact some patients with IBD have a family history of the disease and the prevalence is higher in monozygotic twins than that in dizygotic twins. The prevalence and incidence of IBD vary locally and ethnically. IBD is typically more common in Caucasians in North America and Northern Europe, but very rare in Asians. In recent years, however, the incidence of IBD has markedly increased in Asia [2,3]. According to several epidemiological and clinical studies, the phenotypes and clinical course of IBD differ between Asians and Europeans [4]. In Asia, the incidence of UC is higher than that of CD. In addition, UC patients in Asia have better prognosis than those in Western countries. There is no difference in the prevalence of CD according to sex in the West. However, in Asia, the male to female CD prevalence ratio ranges from 1.67:1 to 2.9:1, with a higher prevalence in men. In Western countries, the rates of ileal, colonic, and ileocolonic diseases are nearly the same among patients with CD. In contrast, in Asia, ileocolonic disease accounts for approximately two-thirds of patients with CD, while colonic disease accounts for only approximately 10% of patients with CD, and the incidence of perianal diseases, such as anal fistula is higher than those of the West. Additionally, extraintestinal manifestations, colorectal cancer, colectomy, and familial aggregation have lower incidence in Asians than those in Westerners. However, it is currently unclear whether Asian-specific clinical features of IBD are entirely due to the differences in environmental factors between Asia and the West, emphasizing the need for genetic studies of IBD in Asian populations. 

Therefore, this review focuses on data from Asian countries to study heredity aspects of IBD and summarize research results. These data might be applicable in the clinical medicine.

## 2. Evidence for Genetic Factors of IBD

From an epidemiologic perspective, because of the familial aggregation of IBD and tendency for the IBD occurrence in Jewish people, particularly Ashkenazi Jewish people, it is thought that IBD is heritable with genetic risk factors in the West. To detect genetic evidence, Kirsner and Spencer conducted a family aggregation study to summarize existing literature in 1963, thus establishing the first report of family history of IBD and subsequent studies supported these predictions [5,6,7]. Familial aggregation does not only refer to genetic factors but also to environmental factors. Determining the contribution of genetic and environmental factors to IBD development is difficult. However, previous studies of twins, couples, and distant family members have suggested that genetic factors are major contributors to familial aggregation [8]. Additionally, UC and, particularly, CD families showed a high concordance in clinical features, such as disease location and extent [9]. 

A study of twins in Europe in 1988 showed that proband concordance in monozygotic twins was 58.3% in CD and 6.3% in UC. In contrast, in dizygotic twins, the proband concordance was 3.9% in CD. There was no concordance in UC. However, concordance was higher in monozygotic twins than that in dizygotic twins [10]. Thus, genetic factors are an important cause of both CD and UC, with stronger effects in CD. According to the results of six twin studies conducted in Europe, the proband concordance of CD was 30.3% in 112 pairs of monozygotic twins and 3.6% in 196 pairs of dizygotic twins. The proband concordance of UC was 15.4% in 143 pairs of monozygotic twins and 3.9% in 206 pairs of dizygotic twins, suggesting that genetic factors could affect the risk of IBD [11,12]. In the West, 5.2–22.5% of patients with CD and 6.6–15.8% of patients with UC had a family history of IBD [13]. The lifetime risk of IBD in first-degree relatives of patients with CD was 7.8% in Jewish people and 4.8–5.2% in non-Jewish people. The lifetime risk of IBD in first-degree relatives of patients with UC was 5.2% in Jewish people and 1.6% in non-Jewish people [7]. In a Danish national epidemiological study performed during 1977–2011, risks of CD onset in first-degree relatives of patients with CD and UC were 7.77, and 4.08, respectively [14]. The familial aggregation of IBD, observed in twin studies, and the increased risk of IBD in proband relatives, have led to studies on molecular genetics of IBD. 

Studies in Asia are less common than those in the West. Previous studies have shown that familial aggregation of IBD is significantly lower in Asia than that in the West, with only 0–6.7% and 0–4.6% of Asian patients having family history of UC, and CD, respectively [3,8,15,16,17,18]. According to a study performed in Korea in 2006, a family history of IBD was observed in 1.88% (UC, 2.01%; CD, 1.51%) of first-degree relatives among 1,440 patients with IBD (1043 patients with UC and 397 patients with CD) [8]. Additionally, the relative risk of IBD after correcting age and sex in first-degree relatives of patients with IBD was 13.8 (first-degree relatives of patients with UC: 13.5; first-degree relatives of patients with CD: 15.1), which was comparable to the risk of 10–15 in the West. This suggests that the prevalence of IBD in first-degree relatives of Korean patients with IBD is 13.8-fold higher than that in the general population. Meanwhile, the lifetime risk of IBD in first-degree relatives of patients with IBD was 0.54% (0.12% in parents, 0.79% in siblings, and 1.43% in children). In comparison, in a Korean study performed in 2016, the proportions of UC and CD in first-degree relatives were 5.1% among 3,266 patients, with UC and 4.8% among 2805 patients with CD, respectively [19]. Although these proportions are still lower in Korea compared to those in Western countries, they are increasing recently. In conclusion, the incidence of family history of IBD, and the lifetime risk of disease development in families with IBD in Asia were lower than those in Western countries. However, this is because the prevalence of IBD itself in Asia, is lower than that in the West, and the relative risk in Asia is similar to that in the West, suggesting that a family history of IBD is an important risk factor in Asian populations.

## 3. History of IBD Genetics

Various studies have been conducted to identify the genes associated with the risk of developing IBD. Early linkage analysis was performed to evaluate IBD familial aggregation; in 1996, the first IBD1 locus, associated with CD, was found on chromosome 16, followed by the discovery of IBD2–IBD9 [20]. In 2001, fine mapping confirmed that nucleotide-binding oligomerization domain containing 2 (*NOD2*)/caspase recruitment domain family member 15 (*CARD15*) of the IBD1 locus was related to CD and three single-nucleotide polymorphisms (SNPs) of *NOD2/CARD15*, rs2066844 (p.Arg702Trp), rs2066845 (p.Gly908Arg), and rs2066847 (p.Leu1007fsinsC), were significantly associated with CD [21]. Genetic linkage analysis is an appropriate method for identifying a small number of loci with high penetrance, and thus is limited to identifying IBD-associated genes. A genome-wide association study (GWAS), based on the so-called, “Common Disease, Common Variant” hypothesis, has been attempted. This hypothesis states that the sum of several mutations with low individual effects together can contribute to the development of relatively common complex diseases such as IBD. 

A GWAS was conducted to evaluate SNPs, distributed in the human genome, by understanding the linkage disequilibrium architecture and development of SNP chips for microarray analysis. GWAS can identify loci and genes, associated with certain diseases, by comparing allele frequencies of numerous SNPs between a large number of patients and controls. The first GWAS for IBD was performed on Japanese patients with CD in 2005 [22]. Tumor necrosis factor superfamily member 15 (*TNFSF15*) was identified as a CD-associated gene, using 72,728 gene-based SNP markers, selected from Japanese SNP database in 484 patients with CD and 1097 controls (odds ratio [OR]: 2.17, P = 1.71 × 10^−14^). This result was replicated in two European IBD cohorts. Next, GWAS was performed mainly in European patients with IBD, and interleukin-23 receptor (*IL23R*), autophagy-related 16-like 1 (*ATG16L1*), 5p13, and 10q21 were identified as CD-related loci [23,24,25,26]. A follow-up GWAS showed that Toll-like receptor 4 (*TLR4*), signal transducer and activator of transcription 3 (*STAT3*), *NKX2-3*, and *CARD9* associated with the innate immune response pathway, *TNFSF15*, protein tyrosine phosphatase non-receptor type 2 (*PTPN2*), *IL12B*, and interferon regulatory factor 5 (*IRF5*) related to the adaptive immune response pathway, and *NOD2/CARD15*, *ATG16L1*, and the immunity-related guanosine triphosphatase M (*IRGM*), related to autophagy and intracellular bacterial handling were associated with CD, improving our understanding of the pathophysiology of CD [12,27,28]. 

GWAS of UC revealed a link between genes associated with epithelial barriers such as hepatocyte nuclear factor 4 alpha (*HNF4A*), cadherin 1 (*CDH1*), and laminin subunit beta-1 (*LAMB1*) and the human leukocyte antigen (HLA) locus and UC [29,30,31]. However, multiple loci revealed by GWAS accounted for only a small part of the heritability of IBD, indicating missing heritability. To address this issue, many studies have been conducted to detect variants with low OR (less than 1.1 or 1.2) by increasing the number of samples by meta-analysis of existing GWAS. A meta-analysis of fifteen GWAS and additional Immunochip data revealed 163 loci related to European lineage IBD (30 loci specific for CD, 23 loci specific for UC, and 110 loci for both diseases), including genes, such as *NOD2* and *ATG16L1* that were correlated with CD only, extracellular matrix protein 1 (*ECM1*) and *IL10* that were correlated with UC only, and *IL23R* that correlated with both diseases [32]. Of 53 disease-specific loci, 43 showed the same directional effect in both CD and UC, suggesting that nearly all biological mechanisms involved in one disease are also involved in the other disease [5,32]. Out of the 163 IBD risk loci identified by GWAS/Immunochip meta-analysis, approximately 70% (113 of 163 loci) of IBD risk loci were shared by other complex diseases or traits and 66 loci were shared by other immune-mediated diseases, such as ankylosing spondylitis and psoriasis [32]. Six (*IL12B*, *IFNGR2*, *STAT1*, *IRF8*, *TYK2*, *STAT3*) of the eight genes, associated with Mendelian susceptibility to mycobacterial disease, were detected in IBD loci and seven of the eight loci related to leprosy were associated with IBD. A recent GWAS meta-analysis of populations with European ancestry has reported more than 240 susceptibility genetic loci for IBD [33]. 

## 4. Genetic Studies in Patients with IBD in Asia

Different clinical features of IBD in Asia, compared to those in the West, are thought to be due to genetic and environmental differences. The first GWAS of IBD was performed on Japanese patients with CD. However, subsequent studies with Asian populations were predominantly replication studies of loci identified in European lineages. 

Genetic studies in Asia have shown that Arg702Trp, Gly908Arg, and Leu1007fsinsC, which are common mutations in *NOD2/CARD15*, are independent of the risk of CD in East Asian populations such as Koreans, Japanese, and Chinese [34,35]. Different correlations of *ATG16L1* and *IL23R*, with IBD, have been detected in studies conducted in Korea, Japan, and China [34,35,36]. Variants in *IL-23R*, *IL17A*, *IRGM*, and *ATG16L1* were associated with CD in Korea, but not in Japan [36,37,38,39]. In China, known variants in *IL23R* were not detected. However, non-synonymous SNP rs11465788 (Gly149Arg) showed an association with CD [40]. In a GWAS analysis of 1535 Japanese patients with CD, two new CD susceptibility loci (rs1487630 at 4p14 and rs7329174 at 13q14 in erythroid-like transcription factor family 1 [*ELF1*]) were reported in addition to previously reported loci in major histocompatibility complex (MHC) regions, *TNFSF15* and *STAT3* (Table 1) [41]. In a GWAS analysis of 2311 Korean patients with CD, six replicated known loci (*TNFSF15*, *IL23R*, three in the MHC region, and *RNASET2-FGFR1OP-CCR6*) among 140 loci identified in the West were discovered in addition to three new loci (rs6856616 at 4p14 in *TBC1D1-KLF3*, rs11195128 at 10q25 in *SMNDC1-DUSP5* and rs11235604 at 11q13 in *ATG16L2*, rs11235667 in *ATG16L2-FCHSD2*) [42]. Subsequent Immuno-chip analysis of IBD in Korea, revealed that six susceptibility loci, reported in Caucasians, were related to CD in Koreans [43]. Thus, the total number of loci related to CD in Koreans increased to 15, explaining 7.27% of total genetic variance of CD risk. In a recent study in Japan, genotype data of 7,934,670 SNPs were analyzed by GWAS using the Japonica array and imputation from 713 Japanese patients with CD and 2082 controls [44]. As a result, rs488200 located upstream of *RAP1A* was found to be significantly associated with CD (OR = 1.31, *p* = 4.36 × 10^−8^). Rap1 has been reported to control the homing of T cells to the colon to prevent colitis onset in a mouse model [45]. Low expression of *RAP1A* increases the risk of CD [44]. These results suggest that Asian CD has unique characteristics compared to Western CD.

In genetic studies of Asian patients with UC, TNF-α-308 polymorphism (Korea, Japan, China and Turkey), cytotoxic T-lymphocyte associated antigen-4 (Japan, China), and MHC-I chain-related genes A alleles (Japan, China) were associated with UC only in Asians, but not in Westerners [35]. An Asian GWAS for UC was performed in Japan in 1384 patients, with UC and 3057 controls in 2009 [47]. It revealed a strong association of UC with MHC regions and three new susceptibility loci (rs1801274 in the immunoglobulin receptor gene FCGR2A, rs17085007 at 13q12 and rs2108225 in the glycoprotein gene SLC26A3). A GWAS in Korea for UC was performed in 2013 [48]. An analysis of 805 cases and 1471 controls showed a strong association of UC with three genetic loci, previously reported in Caucasians (rs9271366 in HLA class II, rs16940186 at 16q24.1, and rs4654903 at 1p36 in RNF186-OTUD3-PLA2G2E). Immunochip analysis of Korean patients with UC revealed 13 susceptibility loci for Korean UC, including the HLA region, accounting for 5.6% of the total genetic variance of UC risk, which was previously found in Western populations [49]. A GWAS conducted in northern Indians in 2015 confirmed that UC susceptibility genes were shared at a certain rate between northern Indians and Europeans [50]. It also identified three HLA-independent risk loci encompassing, *3.8-1*, *BAT2*, *MSH5*, *HSPA1L*, *SLC44A4*, *CFB*, and *NOTCH4*. Therefore, more overlap in associations with Western subjects was observed in UC than that in CD. The number of loci for CD and UC in Asia is lower than that in European populations. The power to explain genetic variance is also lower in Asia.

In 2015, the International IBD Genetics Consortium (IIBDGC) published the results of the first trans-ancestry association study of IBD, including 86,640 European individuals and 9846 East Asian, Indian, and Iranian lineages [51]. As a result, 38 new loci, associated with IBD, such as *ATG4B*, *OSMR*, *LY75*, *CD28*, *CCL20*, *NFKBIZ*, *AHR*, and *NFATC1* were identified. The number of IBD-related loci was increased to 231 independent SNPs in a total of 200 loci. That study also compared the genetic background of IBD between Western and East Asian populations. Although the allele frequency and effect size of polymorphisms differed, the genomic region involved in IBD was mostly common among races. 

Variants identified by GWAS, GWAS meta-analysis, and Immunochip analysis account for less than 30% of the heritability of IBD and missing heritability remains an issue. Therefore, the methods for identifying variants with a minor allele frequency of 0.05 or less, that can greatly influence the etiology of IBD, such as fine mapping by next-generation sequencing (NGS), including whole exome sequencing and whole genome sequencing, are expected to clarify the genetic architecture related to IBD onset. Xu et al. have found that discs large homolog 1 (*DLG1*) is a novel CD susceptibility gene by performing whole exome sequencing in patients with CD in China [52]. Hong et al. have confirmed the association between genetic variants in *TNFSF15*, *IL23R*, and *ATG16L1* and CD risk by deep sequencing of 131 CD-associated genes in Korean patients with CD, and identified eight new CD risk loci [37]. 

An Asian-specific meta-analysis of 4156 IBD cases and 4904 controls and trans-ethnic meta-analysis of 38,155 cases and 48,485 controls in a European population was performed to detect additional IBD susceptibility loci [53]. As a result, three novel susceptibility loci (rs2624435 at 5p15 in *MYO10-BASP1* [IBD], rs57275892 at 14q13 in *PPP2R3C/KIAA0391/PSMA6/NFKB1A* [CD], and rs7170683 at 15q26 in *LRRK1* [CD]) and four novel secondary associations within previously known loci at *NCF4*, *TSPAN32*, *CIITA*, and *VANGL2* were discovered. Comparison of Asian and European populations for the effect sizes from all 193 SNPs known as IBD susceptibility loci and seven new associations identified in this study revealed a positive correlation in the effect direction in both CD and UC (*r*^2^ = 0.40 and *p* = 1.54 × 10^−23^ for CD; *r*^2^ = 0.57 and *p* = 1.84 × 10^−37^ for UC). This was consistent with a previous large-scale study reporting significant genetic overlap between European and East Asian populations [51]. Thus, nearly half of IBD susceptibility loci were shared between Asian and Caucasian races. However, genetic heterogeneities contribute to the risk of IBD in several loci, including *NOD2/CARD15*, *TNFSF15*, *ATG16L1*, *IL23R*, and *IRGM*. The discovery of additional risk variants by NGS may reduce genetic differences in IBD between Asian and Western populations. One point that needs to be considered when comparing differences between Asian and Western genetic researches is that globalization such as migration, travel for school, family, or career has led to a rise in the number of patients that have mixed ancestry. This may also confound or influence observations and results of genetic studies. 

## 5. Susceptibility Genes and Pathophysiology

### 5.1. NOD2/CARD15

Susceptibility genes indicate a functional dysfunction of the intestinal epithelium, mucosal immune system, and resident microbiota as the pathophysiology of IBD. Impaired genetic regulation of the innate immune response to the normal flora is a key mechanism in the etiology of CD and UC as gene mutations, such as *NOD2/CARD15*, *TLR4*, and *CD14* in patients with IBD, can cause abnormalities in the handling of intestinal bacteria. Intestinal microorganisms are recognized by innate immune system cells, particularly pattern recognition receptors, Toll-like receptors (TLRs), and NOD-like receptors that are distributed on the surface, or within epithelial cells, and monocytes/macrophages. Intestinal bacteria and the host conduct cross-talk through TLR and NOD-like receptor. If the receptor contains a genetic defect, recognition of the microbial antigen is dysregulated, resulting in inflammation [54]. *NOD2/CARD15* mutations, particularly Arg702Trp, Gly908Ar, and Leu1007fsinsC SNPs that are functionally linked to deficiencies in antimicrobial defense are highly associated with CD risk in the European lineage, but not in East Asia [35]. In contrast, SNP5 and JW1 mutations in *NOD2* predisposing individuals to other diseases, were detected in patients with CD in India, China, and Malaysia [55]. 

### 5.2. Autophagy Genes

Autophagy refers to the action of digesting and inducing death of several organelles in the cytoplasm. It is involved in aging, growth, neurodegeneration, cancer, and immunity. Autophagy plays an immunological role in killing intracellular microorganisms and purifying the cytoplasm. GWAS studies have showed that mutations in autophagy genes, such as *ATG16L1* and *IRGM* are related to CD [24,26,56]. NOD2 induces autophagy by recruiting ATG16L1 into the cell membrane during bacterial inflow process which requires RIPK2, ATG5, and ATG7 [57]. Dendritic cells in patients with CD, containing *NOD2* or *ATG16L1* variants, are defective in autophagy induction, bacterial handling, and antigen presentation [58]. Such autophagy gene deficiencies can cause abnormalities in the interactions with intestinal flora and lead to excessive pro-inflammatory responses.

The Paneth cell is a specialized secretory cell type, located under the crypt of Lieberkühn in the small intestine [59]. These cells produce a broad repertoire of antimicrobial peptides such as lysozyme and α-defensins to modulate intestinal microbes. They are important mediators of host innate immune responses. In studies investigating whether Paneth cells are affected by specific loci involved in the pathogenesis of CD, an increase in the ratio of abnormal Paneth cells was associated with the number of CD-associated *NOD2* risk alleles [59]. Cumulative levels of *NOD2* and autophagy gene *ATG16L1* T300A risk alleles showed an additional effect on the ratio of abnormal Paneth cells. Abnormal Paneth cells were also associated with a shorter time to disease recurrence in patients with CD who underwent surgery. Different from results in Western patients with CD, *ATG16L1* T300A was not associated with Paneth cell defects in Japanese patients with CD [60]. Among 56 selected SNPs, only *LRRK2* M2397T was significantly correlated with Paneth cell defects (*p* = 3.62 × 10^−4^). In a Western CD cohort, *LRRK2* M2397T was not correlated with Paneth cell defects (*p* = 0.76). *LRRK2* gene known to affect Paneth cell phenotype in Japanese CD was also associated with autophagy. The phenotype of Paneth cells also could predict the prognosis such as postoperative disease recurrence in Japanese CD.

### 5.3. TNFSF15

*TNFSF15* gene is a strong candidate IBD gene that encodes a novel TNF-like factor. *TNFSF15* is expressed in human CD4+, CD8+ T-cells, monocytes/macrophages, dendritic cells, and umbilical vein endothelial cells [61,62,63]. Its expression is promoted by activation of pathogen-related TLRs. *TNFSF15* plays an important role in T cell activation and proliferation. It binds to specific T cell receptors and enhances mucosal CD4+ T cells via synergistic action with IL12 and IL18 [63]. *TNFSF15* can augment cytokine-induced interferon-gamma (IFN-γ) production by CCR9+ mucosal and gut-homing T cells, leading to increased Th1 responses and mucosal inflammation.

Death receptor 3 (DR3, *TNFRSF25*), a major receptor of *TNFSF15*, is upregulated in activated monocytes, NK cells, NKT cells, B-cells, CD4+ T-helper, and CD8+ T-cytotoxic T cells. When TNFSF15 binds to receptors in the presence of TCR stimulation through NF-κB signaling, it stimulates the proliferation and effector function of CD8+ cytotoxic T cells, T-helper 1 (Th1), Th2, and Th17 cells [64]. 

The IBD risk variant of *TNFSF15* can increase the expression level of *TNFSF15* in macrophages, thereby increasing the activity of pattern recognition receptor of MAPK/NF-κB/PI3K signaling and promoting cytokine production [65]. 

### 5.4. IL23R Pathway

Many components of the IL23 pathway such as *IL23R*, *IL12B*, *STAT3*, Janus kinase 2 (*JAK2*), and tyrosine kinase 2 (*TYK2*) are IBD susceptibility genes, known to be associated with CD and UC development [23,66]. *IL23R* and *IL12B* genes play a key role in the development of T-helper cells through Th1 and Th17 pathways, whose final products are IFN-γ and IL17 [67]. IFN-γ and IL17 are elevated in patients with IBD, particularly IFN-γ in patients with CD. IL17 production is increased in both CD and UC. The JAK2 signaling molecule and STAT3 are related to Th1 and Th17 responses and are involved in several cell activation pathways. Thus, defects in the main pathway involved in immune cell activation may be associated with the development of IBD.

### 5.5. IL10 Pathway

The gene that suppresses immunity, rather than activating it, is also related to IBD. IL10 is an immunosuppressive cytokine that inhibits the synthesis of pro-inflammatory cytokines in macrophages and Th1 cells, and suppresses antigen-presenting cells. In a GWAS study of 1,167 patients with UC and 777 healthy controls, SNP rs3024505 immediately adjacent to the *IL10* gene at 1q32.1 showed the most significant association (OR = 1.46, *p* = 1.35 × 10^−12^) [68]. A meta-analysis of six CD GWASs consisting of 6333 cases and 15,056 controls revealed a significant association with rs3024505 (OR = 1.12, *p* = 1.60 × 10^−14^) [69]. 

In IBD that occurs in people at a very young age, a monogenic gene deficiency may be suspected. A mutation in the gene encoding the IL10 receptor, that causes impaired IL10 signaling in infantile-onset or very early-onset IBD (VEO-IBD) (<6 years) has been identified, supporting monogenic gene deficiency [70]. Mutations in *IL10RA* and *IL10RB* genes encoding the IL10R1 and IL10R2 proteins failed to result in STAT3 phosphorylation by IL10 stimulation, although they caused defects in inhibition of the secretion of TNF-α and other proinflammatory cytokines in peripheral-blood mononuclear cells. According to these findings, VEO-IBD (which occurs very early because of severe mutations) and adult-onset IBD (which occurs late as the efficiency of important immunoregulatory pathways was decreased by SNPs) belong to a single disease group that is genetically continuous, rather than distinct diseases in terms of their pathogenetic mechanism. In Asia, *IL10 receptor A* (*IL10RA*) mutations were reported in seven (50%) of 14 Korean VEO-IBD children, and five (38.5%) of 13 Chinese VEO-IBD patients were reported to be positive for *IL10RA* or *IL10RB* mutation [71,72]. Children with *IL10 receptor* mutations also had perianal fistula, showed poor responses to drug therapy, and required early surgical intervention in the first year of life. More than 50 single gene mutations have been identified in children with a phenotype similar to IBD to date, including *XIAP* deficiency, *ADAM17* deletion, and *FOXP3* mutation [73,74,75]. Most single gene defects were expressed in children younger than six years, particularly in those younger than one year old. Therefore, NGS that can identify rare or low-frequency variants, by sequencing all the regions, rather than known variable sites, is an important diagnostic tool for children with early onset IBD, particularly in those with severe disease phenotypes. 

### 5.6. Major Histocompatibility Complex

The MHC region encodes a number of immunological candidates, including classical HLA molecules presenting antigens [76]. GWAS studies have suggested that there are multiple independent associations with IBD in HLA and non-HLA genes in the MHC region [77,78]. However, these studies lacked statistical power. In GWAS/Immunochip meta-analysis, SNP rs6927022, near *HLA-DQA1* in the HLA region, was the most potent UC-associated locus in the genome, and rs9264942 in the HLA-B locus was the most potent CD-associated HLA locus [12,32]. The proportion of IBD variants accounted for by HLA polymorphism, in UC and CD in Asian populations, is significantly higher than those in European populations, with ORs for *HLA-DQA1* and *HLA-DQB1* regions showing significant differences between Western and Asian populations [51]. Goyette et al. analyzed the high-density genotypes of 7406 SNPs in the MHC region of patients with IBD from more than 32,000 European ancestries [79]. As a result, multiple HLA-DRB1 alleles were found to be associated with IBD as well as *HLA-DQA1* and *HLA-DQB1*, suggesting that HLA-DRB1*01:03 might play a major role in both CD and UC. Class Ⅱ HLA variants play a predominant role and heterozygous advantage has been observed in UC, significantly different from CD, which shows relatively equivalent disease risk contributions of class I and class Ⅱ HLA variants. When analyzed according to IBD subtype, HLA-DRB1*01:03 was found to be associated with colonic CD, while HLA-DRB1*07:01 was associated with the absence of colon involvement. Additionally, HLA-DR molecules associated with increased risk of UC and CD showed three-dimensional peptide-binding grooves that differed from those of HLA-DR molecules associated with a decreased risk of UC and CD. This explains that HLA-DRB1*01:03 has a common association with CD and UC, and this allele is involved in determining the immune response of the colon to local flora. These results suggest that adaptive immune responses play an important role in the colonic environment of IBD. 

Although HLA-related pathways may play an important role in Asian IBD, HLA-DRB1*01:03 was not observed in Asians [80,81,82,83]. Previous studies of HLA in Asian IBD showed that HLA-DRB1*15:02 was associated with UC in Japanese and Koreans [84,85,86,87]. A meta-analysis of IBD genetic studies of Japanese subjects demonstrated that HLA-DRB1*04:05 increased the susceptibility to CD but decreased the susceptibility to UC [88]. In recent years, to determine which HLA alleles or amino acids are associated with Asian IBD risk, Han et al. have used imputation technology of 12,568 Koreans and Japanese (3294 patients with CD, 1522 patients with UC, and 7752 controls) and fine-mapped HLA [80]. Their results revealed that amino acid position 37 of HLA-DRβ1 (DRβ1#37) played an important role in the susceptibility to CD (OR = 0.48, *p* = 3.6 × 10^−67^). Previously known association of haplotypes spanning HLA-C*12:02, HLA-B*52:01, and HLA-DRB1*15:02 with UC was confirmed (OR = 4.01, *p* = 1.2 × 10^−28^).

### 5.7. Genetics and Microbiota

The gut microbiota is known to play a critical role in the pathogenesis of IBD. Environmental factors for IBD, such as diet, stress, smoking, breastfeeding, hygiene, infection, vaccination use, and antibiotics are known to cause gut microbial perturbations. In addition, host genetics such as mutations in *NOD2*, *IL23R*, *ATG16L1*, and *IRGM* can affect gut microbiota [89,90].

Germ-free animals do not develop disease in several models of colitis, to induce Paneth cell phenotype susceptible to colitis in the mouse model, besides mutation of the autophagy gene, a norovirus infection of the mouse is required, while genetic variant or enteral pathogens alone is not enough to cause pathogenic changes [91]. *Bacteroides fragilis* produces immunomodulatory molecules such as polysaccharide A, via the outer membrane vesicles and these molecules can protect mice from colitis [92]. Outer membrane vesicles-mediated protection in colitis requires *ATG16L1* and *NOD2* genes. Susceptibility gene’s polymorphism can promote disease through defects that can sense protective signals of microbiome.

Turpin et al. recruited 1561 healthy individuals, of which 270 belonged to 123 families. Nearly one-third of fecal bacterial taxa were found to be inherited [93]. They also identified rs62171178 (nearest gene *UBR3*) associated with *Rikenellaceae*, rs1394174 (*CNTN6*) associated with *Faecalibacterium*, rs59846192 (*DMRTB1*) associated with *Lachnospira*, and rs28473221 (*SALL3*) associated with *Eubacterium*.

The elevated risk of eleven IBD-associated gene mutations in gut bacterial handling genes (*NOD2*, *CARD9*, *IRGM*, *ATG16L1* and *FUT2*) was significantly associated with a decrease in the genus *Roseburia*, acetate-to-butyrate converters, in healthy intestinal microorganisms [89]. 

## 6. Genetic Factors and Disease Course

Not many factors have been identified in predicting the onset and progression of IBD. The results of IBD genetic research could be used to classify the sub-phenotype of IBD in clinical practice and predict disease progression and prognosis of IBD, enabling the identification of patients who may benefit from early aggressive treatment because of their poor prognosis. 

In a European study about the association between *NOD2*/*CARD15*, a typical CD gene, and phenotype of disease, the *NOD2*/*CARD15* polymorphism was found to be associated with the need for early surgical treatment due to stenosis and increased risk of postoperative recurrence [94]. In Asians with CD, P268S mutation of *NOD2*/*CARD15* was associated with disease onset at young ages and ileocecal, and stenotic diseases and the JW1 mutation in *NOD2*/*CARD15* were found to be associated with a trend toward disease onset of younger age and structuring disease of the patient (Table 2) [55,95]. A systematic review and meta-analysis, looking at whether the identified *NOD2* mutations were relevant for predicting complicated disease, showed that a single mutation in *NOD2* had a small effect size, thereby providing insufficient evidence for a clear association with complicated behavior [96]. However, if any *NOD2* mutation was present, the risk of surgery was as high as 58%. *ATG16L1* mutation was associated with stenosis and perianal invasion, and multidrug resistance 1 (*MDR1*) gene polymorphism was associated with drug refractoriness and severe disease progression [97,98]. Additionally, *IL12* mutation was related to the need for early surgery and time to early surgery. 

In a European IBD chip study of 1528 patients with CD, who were followed for more than 10 years, the genes that affected the susceptibility and phenotype of CD were analyzed [107]. *NOD2* mutation was most strongly associated with invasion to the ileum, stenosing, and penetrating behavior, and disease progression accompanying surgery and complications. In addition to *NOD2* mutation, the genes related to developing fistulizing disease were *IL23R*, LOC441108, and *PRDM1*; the genes associated with surgery were *IRGM*, *TNFSF15*, and C13ORF31; and the genes associated with stenosing disease were *JAK2* and *ATG16L1*. 

The IIBDGC performed genotyping using Immunochip array for 19,713 patients with CD, and 14,683 patients with UC at 49 centers in 16 countries in Europe, North America, and Australia to investigate the association between genotype and phenotype [108]. Genotype–phenotype associations for 156,154 genetic variants were also tested. Researchers used information for 193 SNPs and 23 HLA types, known to be associated with IBD, to create a genetic risk score. Next, they used this score to investigate genetic relationships of ileal CD, colonic CD, and UC. Three gene loci (*NOD2*, MHC, *MST1* 3p21) were associated with the sub-phenotype of IBD, mainly with the location of disease and little or no genetic association with dramatically changing disease behavior over time. Thus, *NOD2*/*CARD15* was associated mainly with disease location (ileum), not with the stenotic phenotype. The predictive models, based on the genetic risk score clearly distinguished colonic CD from ileal CD, and IBD patients, were better classified into the following three groups based on gene risk scores: ileal CD, colonic CD, and UC rather than CD and UC. The CD vs. UC risk score for colonic CD was between CD and UC. Genetically, colonic CD was closer to UC than to purely ileal CD. 

Data from the Immuno-chip project of the IIBDGC, which included 17,379 CD cases, 13,458 UC cases and 22,442 controls, in 15 European countries, were used to determine whether genetic risk scores were helpful for diagnosing IBD [109]. To conduct a risk assessment for CD and UC, researchers constructed an optimal step-wise risk prediction model, using an advanced machine-learning technique. The final predictive model achieved an area under the curve (AUC) of 0.864 and 0.830 for CD and UC, respectively. In a recent study of 409,258 participants with genetic and clinical data in the UK Biobank database, researchers used the LDPred computational algorithm to create a genome-wide polygenic risk score from all variants found in large GWASs involving coronary artery disease, atrial fibrillation, type 2 diabetes, IBD and breast cancer [110]. As a result, the risk score showed that 3.2% of people had a 3-fold higher risk of IBD

To investigate the association between TNFSF15 genotype (demonstrated by CD susceptibility gene) and clinical course of CD, a total of 906 Korean patients with CD having the *TNFSF15* genotype and clinical information were evaluated [99]. Among *TNFSF15* SNPs, non-risk allele homozygotes of rs6478108 CC genotype and rs4574921 CC genotype were found to be independent genetic predictors of stricture/non-perianal penetrating complication and perianal fistula, respectively. To confirm whether *TNFSF15* was useful for stratifying disease behavior in patients with CD, 108 Taiwanese patients with IBD (55 cases of CD and 53 cases of UC) were analyzed [111]. As a result, when genetic marker *TNFSF15* (rs4263839) and serum marker ASCA IgA were combined, the AUC for stricture/perforated phenotype prediction was increased from 0.61 to 0.70 compared to ASCA IgA alone. 

FOXO3 is a member of the FOXO family of transcription factors, most of which are widely expressed to regulate a variety of cellular programs that can affect longevity, including cell-cycle control, DNA repair, and oxidative stress responses [112]. Lee et al. have shown that the minor (G) allele in *FOXO3A* non-coding polymorphism (rs12212067: T > G), which is not related to disease susceptibility, is associated with a mild clinical course of CD in European ancestry cohorts [113,114]. The minor (G) allele has been shown to inhibit the inflammatory response of monocytes through the FOXO3-driven pathway, thus decreasing the production of proinflammatory cytokines, including TNFα through TGFβ1, while increasing the production of anti-inflammatory cytokines, including IL10.

Organic cation/carnitine transporter (OCTN) is a transporter belonging to the solute carrier (SLC) 22A sub-family. In humans, it has two subtypes, OCTN1 and OCTN2. L503F variant of *OCTN1* and g.–207G>C variant of promoter of *OCTN2* have been reported to be significantly correlated with susceptibility and clinical symptoms of CD [115,116]. However, polymorphisms in *OCTN1* and *OCTN2* were reported to be absent in Chinese [117]. A recent study has been performed to identify genetic variants in the *OCTN2* promoter and investigate the function of each variant in Korean patients with CD [102]. As a result, H3, one of three major promoter haplotypes identified for OCTN2, showed a significant decrease in promoter activity. In addition, two polymorphisms in H3 (g.–1889T>C and g.–945T>G) significantly reduced the activity of *OCTN2* promoter. Transcription factors NF-E2 and YY1 were involved in this regulatory mechanism. The correlation analysis with CD showed that functional genetic variant of the *OCTN2* promoter was not related to CD susceptibility. However, haplotype HC, which decreased *OCTN* promoter activity, was significantly correlated with the penetrating pattern and high frequency of surgery in CD patients’ clinical courses. 

To determine the effect of genetic variants on disease prognosis, a within-cases GWAS was conducted using two cohorts of patients with CD from the UK IBD Genetics Consortium and New Zealand [118]. Researchers identified four important loci (*XACT*, MHC, *FOXO3*, *IGFBP1*) that contribute to the prognosis of CD. However, none of them was related to disease susceptibility. Thus, disease initiation is not only differentiated temporally from disease with active symptoms, but also regulated by different genetics and possibly by different biology.

Fewer genetic predictive factors have been identified for UC than for CD. The HLA-DRB1*01:03 allele is associated with extensive disease and colonic resection. However, its frequency is low, thus limiting its clinical usefulness. An attempt has been made to predict refractory UC, using SNPs identified through a GWAS. When the UC Immuno-chip dataset of IIBDGC was used, rs2403456 at 11p15.3 was associated with medically refractory UC and early colectomy due to severe disease (OR 2.15, *p* = 1.22 × 10^−6^) [119,120]. 

Lee et al. have recently conducted an Immuno-chip-based study of 1,961 Korean patients with UC, using an extreme phenotype approach to identify genetic variants affecting the prognosis of UC [105]. Based on the finding that the only significant (*p* < 1 × 10^−4^) association was observed in HLA, researchers focused on HLA sites. HLA imputation data of 243 cases with poor prognosis that required administration of anti-TNF agents and/or colectomy and 364 cases with good prognosis that did not require corticosteroids, thiopurines, anti-TNF agents, or colectomy in three independent discovery cohorts were used to analyze these associations. Replication studies were performed for 145 cases with poor prognosis and 129 cases with good prognosis. As a result, rs9268877 located between HLA-DRA and HLA-DRB showed a significant correlation with poor prognosis with genome-wide significance (OR = 1.72, *p* = 1.04 × 10^−8^). As rs9268877 affected clinical outcomes, the cumulative probability of colectomy over 30 years was significantly higher in homozygotes (AA) than that in non-carriers (GG) (26.3% vs. 6.4%, *p* = 3.30 × 10^−7^). The presence of the risk allele of rs9268877 showed a sensitivity of 80.0% and a specificity of 38.1% for colectomy. These results suggest that genetic variants associated with the prognosis of UC differ from genetic contribution to disease susceptibility. 

## 7. Pharmacogenetics in IBD

### 7.1. Thiopurine

Thiopurine drugs, which include azathioprine (AZA) and its metabolite 6-mercaptopurine (6-MP), have cytotoxic and immunosuppressive activities. Thus, they are widely used for treating several autoimmune diseases, including refractory or chronic active IBD. However, thiopurine drugs can cause adverse events such as myelosuppression, hepatotoxicity, and pancreatitis, of which myelosuppression is the most fatal side effect. Myelosuppression occurs in approximately 2–7% of patients in Western countries [121]. Three important enzymes, thiopurine S-methyltransferase (TPMT), xanthine oxidase, and hypoxanthine-guanine phospho-ribosyl transferase, are competitively involved in the process of in vivo conversion of AZA/6-MP into inactive and active metabolites through complex processes. Decreased TPMT activity associated with *TPMT* allele polymorphism can significantly affect the efficacy and toxicity of AZA/6-MP [122]. Although TPMT activity is less likely to be reduced in Asian patients, leukopenia occurs in 31–56% of patients even at AZA doses that are lower than those in Western countries [123]. 

In an Asian study of genetic susceptibility to side effects of thiopurine, immune-chip analysis of 978 Korean CD patients taking thiopurine was performed [124]. As a result, when a non-synonymous SNP, the presence of rs116855232 (encoding p.Arg139Cys) in nucleoside diphosphate-linked moiety X-type motif 15 (*NUDT15*) was significantly associated with the development of early leukocytopenia, decrease in white blood cells to less than 3,000 cells/mm^3^ within 8 weeks of thiopurine treatment (OR: 35.63, *p* = 4.88 × 10^−94^). The heterozygous (CT) genotype, with one copy of the *NUDT15* risk allele was present in 68.2% of patients with early-onset leukopenia, while the homozygous (TT) genotype was present in 21.2% of such patients. However, there was no homozygous (TT) genotype for the risk allele in controls. Non-carrier (CC) was present in 93.2% of controls. The sensitivity and specificity of this variant for predicting early leukopenia by thiopurine in Koreans were 89.4%, and 93.2%, respectively, with an AUC of 0.92. According to *NUDT15* genotypes, 1.4% of all patients were homozygous (TT), 18.0% were heterogynous (CT), and 80.0% were non-carrier (CC). For the homozygous (TT) genotype, 100% of patients showed early leukopenia. The *NUDT15* risk allele is much more common in Asians than in those of European descent, with rates of 10.4% in Koreans, 7% in Japanese, 13% in Chinese, and 2% in the admixed US population. Although this risk allele was present at a very low frequency in European populations, those with the *NUDT15* risk allele showed a greater risk of leukopenia during thiopurine treatment. Thus, the *NUDT 15* genotype test may be helpful for identifying patients at risk of early leukopenia before thiopurine treatment. Four *NUDT15* coding mutations (p.Arg139Cys, p.Arg139His, p.Val18Ile, and p.Val18_Val19insGlyVal) have been identified. They resulted in 74.4–100% loss of nucleotide diphosphatase activity because *NUDT15* polymorphism could affect thiopurine metabolism [125]. 

When genotypes of *NUDT15* codon 139 were analyzed in Japan, researchers confirmed the association of *NUDT15* p.Arg139Cys with thiopurine-induced leukopenia and alopecia [126]. The dose of thiopurine was lower, and leukopenia was shorter at the time of diagnosis where severe leukopenia were present in patients with genotypes Arg/Cys (RC) and Cys/Cys (CC), compared to those with Arg/Arg (RR) genotype. There was no significant difference in AUCs between genotypes of *NUDT15* codon 139 and *NDUT15* diplotypes (a combined analysis of the haplotypes carrying additional variants) for predicting severe adverse events such as acute severe leukopenia and severe alopecia (AUC for acute severe leukopenia: 0.916 with NUDT15 codon 139 and 0.921 with NDUT15 diplotypes; AUC for severe alopecia: 0.990 with NUDT15 codon 139 and 0.991 with NDUT15 diplotypes)

A meta-analysis of studies conducted in Korea, Japan, Thai, China, and India that involved 1138 patients with CD, acute lymphocytic leukemia or IBD revealed that 311 (27.3%) patients had the *NUDT15* 415T allele [127]. T carriers of *NUDT15* c.415C>T were significantly correlated with the incidence of thiopurine-induced leukopenia (CT + TT vs. CC: risk ratio [RR] = 3.79, *p* < 0.00001; CT vs. CC: RR = 3.41, *p* < 0.00001). This correlation was particularly strong in TT patients and significantly increased by 6.54-fold compared to that in CC patients (TT vs. CC: RR = 6.54, *p* < 0.00001). 

To investigate the effect of *TPMT* pharmacogenetics on thiopurine treatment response in IBD, a randomized controlled trial of 783 IBD patients in 30 Dutch centers, the Thiopurine response optimization by pharmacogenetic testing in inflammatory bowel disease clinics (TOPIC) trial was performed [128]. The effect of *TPMT* genotype-guided thiopurine administration on hematologic adverse drug reactions, such as leukopenia and thrombocytopenia, between genotyped and non-genotyped arms was not significantly different (frequency: 7.4% versus 7.9%; relative risk: 0.93). There was no significant difference in clinical outcome according to disease activity score between the two groups either. However, a subgroup analysis, comparing only *TPMT* variants carriers between the two groups, showed that pharmacogenetic approaches could significantly reduce the risk of hematologic adverse drug reactions in carriers of at least one genetic variant (frequency: 2.6% vs. 22.9%, relative risk: 0.11). 

Because many patients with wild-type *TPMT* and *NUDT15* clinically experience myelosuppression by thiopurine, many cases of thiopurine-induced leukopenia cannot be explained by genetic variants in *TPMT* or *NUDT15*. Kim et al. have reported that fat mass and an obesity-associated gene (*FTO*) p.Ala134Thr (rs79206939) are associated with thiopurine-induced leukopenia according to high-throughput targeted sequencing for GWAS and fine mapping of 1,098 patients with IBD in Korea and Japan. This was also validated in two replication cohorts (OR = 4.3, *p* = 1.3 × 10^−8^) [129]. The frequency of *FTO* p.Ala134Thr is 5.1% in Koreans but less than 0.1% in Western populations. FTO showed nucleotide demethylase activity. In the nucleotide demethylase assay, *FTO* p.Ala134Thr reduced FTO activity by 65% compared to wild-type *FTO*. 

Pancreatitis is one of side effects associated with thiopurine. It occurs in 4–7% of patients with IBD due to hypersensitivity not related to the dose [130,131]. To identify genetic markers for predicting the occurrence of pancreatitis within 3 months of thiopurine administration in patients with IBD, GWAS was carried out for 172 patients and 2,035 controls at 168 institutions worldwide, and replication was performed on an additional 78 patients and 472 controls for validation [132]. As a result, an association was detected within the class II HLA region. The most significant association was identified in rs2647087 (OR 2.59, *p* = 2 × 10^−16^), which was validated in an independent set. Clinically, those with homozygous risk allele at rs2647087 showed an approximately 17% risk of pancreatitis while the risk was 9% in rs2647087 heterozygotes. Therefore, genetic information can be used in a screening test to reduce severe adverse events associated with myelosuppression or pancreatitis before administering thiopurine. 

### 7.2. Anti-TNF Treatment

Genetic variants can affect drug responses, and genetic markers can help select a personalized treatment strategy for individual patients. As some IBD patients do not show response to anti-TNF drugs, several attempts have been made to identify genetic factors for predicting the response of patients to anti-TNF drugs [133,134,135,136,137]. However, earlier studies showed inconsistent results for TNF-α polymorphisms and infliximab (IFX) response, with no association observed between *NOD2*/*CARD15* and IFX non-response. In a retrospective study of 90 moderate-to-severe patients with UC treated with IFX induction therapy, those who were homozygous for IBD risk-increasing *IL23R* variants (rs1004819, rs2201841, rs10889677, rs11209032, rs1495965) showed a significantly higher probability of responding to IFX at 14 weeks than those who were homozygous for risk-decreasing *IL23R* variants (rs7517847, rs10489629, rs11465804, rs1343151) (74.1% versus 34.6%, *p* = 0.001) [138]. In a study to identify predictors of anti-TNF therapy in pediatric patients IBD, 22 (23.4%) of 94 patients (20 patients with UC and 74 patients with CD) showed primary non-responsiveness to IFX [139]. When three pharmacogenetic GWAS loci (rs975664 at 2p12 in *TACR1*, rs4855535 at 3p14 in *FAM19A4*, rs6100556 at 20q13 in *PHACTR3*), previously identified susceptibility locus (rs2836878 at 21q22 in *BRWD1*), pANCA, and diagnosis of UC versus CD were all applied to the predictive model for primary unresponsiveness. It was the most predictable, with a sensitivity of 95%, a specificity of 88%, an accuracy of 92%, a positive likelihood ratio of 8, R^2^ of 0.82, and AUC of 0.98. 

A meta-analysis of 15 studies (including one Japanese study as an Asian study) investigated the association between anti-TNF therapy and polymorphism to identify genetic biomarkers related to anti-TNF therapy in patients with IBD, five different genotypes (*TLR2* rs4696480 TT, *TLR2* rs11938228 AC, *IL6* rs10499563 TT, *IL12B* rs3212227 AC, *IL12B* rs3212217 CG) were significantly correlated with a non-response to anti-TNF in UC [140,141]. Urabe et al. have evaluated associations of SNPs in candidate genes in the IL17 signaling pathway to identify genes related to IFX responses and biomarkers, that could predict IFX effects in 103 Japanese patients, with CD [142]. In multivariate logistic regression analysis, the GG genotype of rs766748 in *IL17F* and CC or CA genotype of rs1883136 in *TRAF3IP2* independently contributed to drug response after one year of IFX treatment in addition to the combined use of immunomodulators and penetrating disease. Matsuoka et al. have studied eight SNPs in 121 Japanese patients with CD who were treated with IFX for more than 1 year by real-time PCR (qPCR) [143]. As a result, the TNF-α 857C>T CC genotype (rs1799724), shorter duration, absence of double dosing, and combination therapy, with immunomodulators were significantly associated with remission in IFX maintenance therapy. 

In recent years, in the Personalizing Anti-TNF therapy in CD (PANTS) cohort, a GWAS was conducted to identify genetic variants associated with anti-drug antibody development in 1240 CD patients in the UK [144]. HLA-DQA1*05 present in approximately 40% of Europeans significantly increased the rate of anti-drug antibody development (hazard ratio, 1.90, *p* = 5.88 × 10^−13^). This immunogenicity was consistent regardless of whether patients were treated with adalimumab or IFX and monotherapy or combination with immunomodulators. 

## 8. Conclusions

The incidence of IBD is increasing worldwide. Many studies have evaluated its pathophysiology and the development of new therapies. Genes associated with CD and UC have been identified. Through studies of their functions, there has been much effort to understand associations among genes, environment, gut microbiota, and immune response in IBD. Such studies have revealed more than 240 loci/genes related to IBD. However, it is estimated that less than 30% of IBD susceptibility can be explained by genetic contributions. Most genes generally have common susceptibility to IBD while a few are specific to CD or UC. Although risk loci for IBD typically overlap between Asians and Westerners, there are genetic heterogeneities in many loci/genes, such as *NOD2/CARD15*, *TNFSF15*, and HLA that contribute to the risk of IBD. 

Genetic studies have revealed key insights into the pathophysiology of IBD, including innate and adaptive immunity, autophagy, defective bacterial handing, IL23 and IL10 signaling, and so on. They might lead to new treatments for IBD. Although genetic studies are not universally applicable in the clinical field, they might be useful for diagnosing and categorizing IBD, predicting therapeutic responses to drugs and toxicity, and assessing prognosis through risk modeling. If the problem of missing heritability can be solved by NGS or epigenetics approaches such as methylation and microRNA analyses in addition to existing GWAS and Immunochip analysis, biomarkers for IBD can be identified and more detailed precision medicine may be provided to individual patients. 

## Figures and Tables

**Table 1:** Genome-wide association and Immunochip studies on inflammatory bowel disease susceptibility genes in Asia [46].

Disease	Country	Study	New SNP	Positional Candidate Gene or Region	Replicated Gene or Region	Reference
CD	Japan	GWAS	rs1487630	4p14	MHC	[41]
			rs7329174	*ELF1*	*TNFSF15*	
					*STAT3*	
	Korea	GWAS	rs6856616	*TBC1D1-KLF3*	*TNFSF15*	[42]
			rs11195128	*SMNDC1-DUSP5*	*IL23R*	
			rs11235604	*ATG16L2*	MHC	
			rs11235667	*ATG16L2-FCHSD2*	*RNASET2-FGFR10P-CCR6*	
	Korea	Immunochip			*GPR35*	[43]
					*ZNF365*	
					*ZMIZI*	
					*NKX2-3*	
					*PTPN2*	
					*USP25*	
	Japan	GWAS	rs488200	*RAP1A*	*TNFSF15*	[44]
					*HLA-DQB1*	
					4p14	
					*ZNF365*	
					*IL12B*	
					*IL27*	
					*IL23R* G149R	
UC	Japan	GWAS	rs1801274	*FCGR2A*	*JAK2*, *INSL6, INSL4*	[47]
			rs17085007	13q12	MHC	
			rs2108225	*SLC26A3*		
	Korea	GWAS			MHC	[48]
					16q24.1	
					*RNF186-OTUD3-PLA2G2E*	
	Korea	Immunochip			*IL23R*	[49]
					*IRF5*	
					*(JAK2)*	
					*(TNFRSF14)*	
					*(IL10*-*1L19)*	
					*TNFSF15*	
					*(UBE2L3*-*YDJC)*	
					*FCGR2A*	
					*(GPR12*-*USP12)*	
	India	GWAS	rs2261033	*BAT2*	*PTPRC*	[50]
			rs2736428	*SLC44A4*	MHC	
			rs2075800	*HSPA1L*	*TAP2*	
			rs549182	*NOTCH4*		
			rs4151657	*CFB*		
			rs3749946	*3.8-1*/*HCG26*		
			rs707939	*MSH5*		

CD = Crohn’s disease, UC = ulcerative colitis, GWAS = genome-wide association study, MHC = major histocompatibility complex, () = denotes nearby genes.

**Table 2:** Genetics and disease course in Asian studies [35].

Disease	Clinical Phenotype	Genetic Variants	Country	References
CD	Structuring disease	*NOD2/CARD15* (P268S mutation)	China	[95]
		*NOD2/CARD15* (JW1 mutation)	China, Malaysia	[55,95]
		*TNFSF15* rs6478108 CC	Korea	[99]
	Penetrating disease	*CTLA-4*	Japan	[100]
		*LTA* Thr60Asn	Korea	[101]
		*TNFSF15* rs6478108 CC	Korea	[99]
		*OCTN2* promoter haplotype HC	Korea	[102]
	Perianal lesions	*TNFSF15* rs4574921 CC	Korea	[99]
		*TNFSF15* rs6478109 C	Japan	[103]
		*TNFSF15* rs7848647 G	Japan	[103]
		*TNFSF15* rs4979462 A	Japan	[103]
UC	Severe disease	*IL8* haplotype -353A/-251A/+678T	China	[104]
		HLA-DRA-HLA-DRB rs9268877 A	Korea	[105]
	Pancolitis	*MTHFR* 677TT	China	[106]
